# 4-{(*Z*)-2-[(*E*)-Benzyl­idenehydrazinyl­idene]-3,6-dihydro-2*H*-1,3,4-thia­diazin-5-yl}-3-phenyl-1,2,3-oxadiazol-3-ium-5-olate

**DOI:** 10.1107/S1600536811010609

**Published:** 2011-03-26

**Authors:** Hoong-Kun Fun, Ching Kheng Quah, Nithinchandra, Balakrishna Kalluraya

**Affiliations:** a X-ray Crystallography Unit, School of Physics, Universiti Sains Malaysia, 11800 USM, Penang, Malaysia; b Department of Studies in Chemistry, Mangalore University, Mangalagangotri, Mangalore 574 199, India

## Abstract

The title compound, C_18_H_14_N_6_O_2_S, exists in *trans* and *cis* configurations with respect to the two acyclic C=N bonds [bond lengths = 1.2835 (9) and 1.3049 (9) Å]. The 3,6-dihydro-2*H*-1,3,4-thia­diazine ring adopts a half-boat conformation. The oxadiazol-3-ium ring makes dihedral angles of 53.70 (4) and 60.26 (4)° with the two phenyl rings. In the crystal, mol­ecules are linked *via* pairs of inter­molecular N—H⋯N hydrogen bonds, generating *R*_2_^2^(8) ring motifs, and are further linked *via* inter­molecular C—H⋯O and C—H⋯S hydrogen bonds into a three-dimensional network. The short inter­molecular distance between the oxadiazol-3-ium rings [3.4154 (4) Å] indicates the existence of a π–π inter­action.

## Related literature

For general background to and the biological activity of sydnone derivatives, see: Newton & Ramsden (1982 ▶); Wagner & Hill (1974 ▶); Kalluraya & Rahiman (1997 ▶). For the preparation, see: Kalluraya *et al.* (2003 ▶). For the stability of the temperature controller used in the data collection, see: Cosier & Glazer (1986 ▶). For bond-length data, see: Allen *et al.* (1987 ▶). For hydrogen-bond motifs, see: Bernstein *et al.* (1995 ▶). For ring conformations, see: Cremer & Pople (1975 ▶).
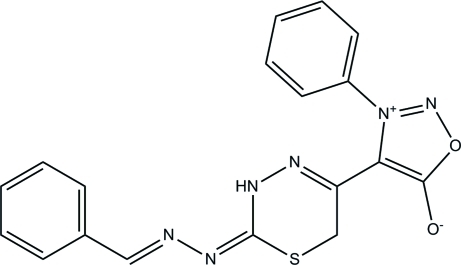


## Experimental

### 

#### Crystal data

C_18_H_14_N_6_O_2_S
*M* *_r_* = 378.41
Triclinic, 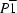

*a* = 6.8752 (2) Å
*b* = 10.1335 (3) Å
*c* = 12.7374 (4) Å
α = 78.578 (1)°
β = 88.984 (1)°
γ = 85.874 (1)°
*V* = 867.58 (5) Å^3^
*Z* = 2
Mo *K*α radiation
μ = 0.21 mm^−1^
*T* = 100 K
0.58 × 0.27 × 0.08 mm

#### Data collection

Bruker SMART APEXII DUO CCD area-detector diffractometer
Absorption correction: multi-scan (*SADABS*; Bruker, 2009 ▶) *T*_min_ = 0.885, *T*_max_ = 0.982
29236 measured reflections
7567 independent reflections
6818 reflections with *I* > 2σ(*I*)
*R*_int_ = 0.022

#### Refinement

*R*[*F* ^2^ > 2σ(*F* ^2^)] = 0.033
*wR*(*F* ^2^) = 0.101
*S* = 1.03
7567 reflections
248 parameters
H atoms treated by a mixture of independent and constrained refinement
Δρ_max_ = 0.65 e Å^−3^
Δρ_min_ = −0.37 e Å^−3^

### 

Data collection: *APEX2* (Bruker, 2009 ▶); cell refinement: *SAINT* (Bruker, 2009 ▶); data reduction: *SAINT*; program(s) used to solve structure: *SHELXTL* (Sheldrick, 2008 ▶); program(s) used to refine structure: *SHELXTL*; molecular graphics: *SHELXTL*; software used to prepare material for publication: *SHELXTL* and *PLATON* (Spek, 2009 ▶).

## Supplementary Material

Crystal structure: contains datablocks global, I. DOI: 10.1107/S1600536811010609/sj5123sup1.cif

Structure factors: contains datablocks I. DOI: 10.1107/S1600536811010609/sj5123Isup2.hkl

Additional supplementary materials:  crystallographic information; 3D view; checkCIF report

## Figures and Tables

**Table 1:** Hydrogen-bond geometry (Å, °)

*D*—H⋯*A*	*D*—H	H⋯*A*	*D*⋯*A*	*D*—H⋯*A*
N3—H1*N*3⋯N2^i^	0.852 (15)	2.015 (15)	2.8664 (9)	178.3 (11)
C14—H14*A*⋯O2^ii^	0.93	2.58	3.2303 (10)	127
C15—H15*A*⋯O2^iii^	0.93	2.51	3.2391 (9)	136
C18—H18*A*⋯S1^iv^	0.93	2.84	3.7061 (8)	155

Symmetry codes: (i) 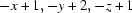; (ii) 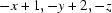; (iii) 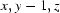; (iv) 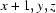.

**Fig. 1.**
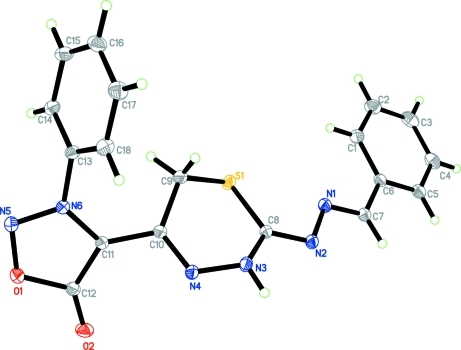
The molecular structure of the title compound showing 50% probability displacement ellipsoids for non-H atoms and the atom-numbering scheme.

**Fig. 2.**
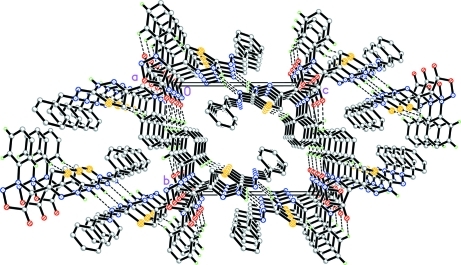
The crystal structure of the title compound, viewed along the a axis. H atoms not involved in hydrogen bonds (dashed lines) have been omitted for clarity.

## supplementary crystallographic information

### Comment

Sydnones are a class of mesoionic compounds containing a 1,2,3-oxadiazole ring system. A number of sydnone derivatives have shown diverse biological activities such as anti-inflammatory, analgesic and anti-arthritic (Newton & Ramsden, 1982; Wagner & Hill, 1974) properties. Sydnones with heterocyclic substituents at the 4-position are also known to exhibit a wide range of biological properties (Kalluraya & Rahiman, 1997). Encouraged by these reports and in continuation of our research for biologically-active nitrogen-containing heterocycles, a thiadiazine moiety was introduced at the 4-position of the phenylsydnone. A series of thiadiazines were synthesized by the condensation of 4-bromoacetyl-3-arylsydnones with N'-(phenylmethylidene)carbonohydrazide. 4-Bromoacetyl-3-arylsydnones were in turn obtained by the photochemical bromination of 4-acetyl-3-arylsydnones (Kalluraya *et al.*, 2003).

The molecular structure is shown in Fig. 1. Bond lengths (Allen *et al.*, 1987) and angles are within normal ranges. The title compound exists in *trans* and *cis* configurations with respect to the acyclic C7═N1 and C8═N2 bonds [C7═N1 = 1.2835 (9) Å and C8═N2 = 1.3049 (9) Å]. The 3,6-dihydro-2H-1,3,4-thiadiazine ring (S1/N3/N4/C8-C10) adopts a half boat-conformation with atom C9 deviating by 0.359 (1) Å from the mean plane through the remaining atoms, puckering parameters (Cremer & Pople, 1975) Q = 0.5575 (7) Å, Θ = 108.09 (7)° and φ = 137.99 (7)°. The oxadiazol-3-ium ring (O1/N5/N6/C11/C12) makes dihedral angles of 53.70 (4) and 60.26 (4) ° with two phenyl rings (C1-C6 and C13-C18).

In the crystal packing (Fig. 2), the molecules are linked *via* pairs of intermolecular N3–H1N3···N2 hydrogen bonds (Table 1), generating R_2_^2^(8) ring motifs (Bernstein *et al.*, 1995) and are further linked *via* intermolecular C14–H14A···O2, C15–H15A···O2 and C18–H18A···S1 hydrogen bonds (Table 1) into a three-dimensional network. The crystal packing is further consolidated by π-π stacking interactions between the centroids of O1/N5/N6/C11/C12 (Cg1) rings, with Cg1···Cg1^v^ distance of 3.4154 (4) Å [symmetry code: (v) 2-X, 2-Y, -Z].

### Experimental

To a solution of 4-bromoacetyl-3-(p-anisyl)sydnone (0.01 mol) and *N*'-(phenylmethylidene) carbonohydrazide (0.01 mol) in ethanol, catalytic amount of anhydrous sodium acetate was added. The solution was stirred at room temperature for 2 to 3 h. The solid product that separated out was filtered and dried. It was then recrystallized from ethanol. Crystals suitable for X-ray analysis were obtained from 1:2 mixtures of DMF and ethanol by slow evaporation.

### Refinement

H1N3 was located in a difference Fourier map and allowed to refined freely. The remaining H atoms were positioned geometrically and refined using a riding model with C–H = 0.93 or 0.97 Å and *U*_iso_(H) = 1.2 *U*_eq_(C). The highest residual electron density peak is located at 0.68 Å from C13 and the deepest hole is located at 0.71 Å from S1.

### Crystal data

**Table:**

C_18_H_14_N_6_O_2_S	*Z* = 2
*M**_r_* = 378.41	*F*(000) = 392
Triclinic, *P*1	*D*_x_ = 1.449 Mg m^−^^3^
Hall symbol: -P 1	Mo *K*α radiation, λ = 0.71073 Å
*a* = 6.8752 (2) Å	Cell parameters from 9935 reflections
*b* = 10.1335 (3) Å	θ = 2.9–37.5°
*c* = 12.7374 (4) Å	µ = 0.21 mm^−^^1^
α = 78.578 (1)°	*T* = 100 K
β = 88.984 (1)°	Block, yellow
γ = 85.874 (1)°	0.58 × 0.27 × 0.08 mm
*V* = 867.58 (5) Å^3^	

### Data collection

**Table:**

Bruker SMART APEXII DUO CCD area-detector diffractometer	7567 independent reflections
Radiation source: fine-focus sealed tube	6818 reflections with *I* > 2σ(*I*)
graphite	*R*_int_ = 0.022
φ and ω scans	θ_max_ = 35.0°, θ_min_ = 1.6°
Absorption correction: multi-scan (*SADABS*; Bruker, 2009)	*h* = −11→11
*T*_min_ = 0.885, *T*_max_ = 0.982	*k* = −16→16
29236 measured reflections	*l* = −20→20

### Refinement

**Table:**

Refinement on *F*^2^	Primary atom site location: structure-invariant direct methods
Least-squares matrix: full	Secondary atom site location: difference Fourier map
*R*[*F*^2^ > 2σ(*F*^2^)] = 0.033	Hydrogen site location: inferred from neighbouring sites
*wR*(*F*^2^) = 0.101	H atoms treated by a mixture of independent and constrained refinement
*S* = 1.03	*w* = 1/[σ^2^(*F*_o_^2^) + (0.0601*P*)^2^ + 0.2223*P*] where *P* = (*F*_o_^2^ + 2*F*_c_^2^)/3
7567 reflections	(Δ/σ)_max_ = 0.001
248 parameters	Δρ_max_ = 0.65 e Å^−^^3^
0 restraints	Δρ_min_ = −0.37 e Å^−^^3^

### Special details

**Table:**

Experimental. The crystal was placed in the cold stream of an Oxford Cyrosystems Cobra open-flow nitrogen cryostat (Cosier & Glazer, 1986) operating at 100.0 (1) K.
Geometry. All esds (except the esd in the dihedral angle between two l.s. planes) are estimated using the full covariance matrix. The cell esds are taken into account individually in the estimation of esds in distances, angles and torsion angles; correlations between esds in cell parameters are only used when they are defined by crystal symmetry. An approximate (isotropic) treatment of cell esds is used for estimating esds involving l.s. planes.
Refinement. Refinement of F^2^ against ALL reflections. The weighted R-factor wR and goodness of fit S are based on F^2^, conventional R-factors R are based on F, with F set to zero for negative F^2^. The threshold expression of F^2^ > 2sigma(F^2^) is used only for calculating R-factors(gt) etc. and is not relevant to the choice of reflections for refinement. R-factors based on F^2^ are statistically about twice as large as those based on F, and R- factors based on ALL data will be even larger.

### Fractional atomic coordinates and isotropic or equivalent isotropic displacement parameters (Å^2^)

**Table:**

	*x*	*y*	*z*	*U*_iso_*/*U*_eq_	
S1	0.30498 (3)	0.783931 (19)	0.313847 (14)	0.01687 (5)	
O1	0.78647 (9)	1.04039 (5)	−0.06087 (4)	0.01590 (10)	
O2	0.64969 (9)	1.17463 (5)	0.04865 (5)	0.01895 (11)	
N1	0.14753 (10)	0.81808 (6)	0.50454 (5)	0.01503 (11)	
N2	0.31270 (10)	0.89086 (6)	0.48880 (5)	0.01459 (11)	
N3	0.57604 (10)	0.93168 (6)	0.37674 (5)	0.01437 (10)	
N4	0.65689 (9)	0.96382 (6)	0.27631 (5)	0.01327 (10)	
N5	0.83490 (10)	0.90536 (6)	−0.05820 (5)	0.01491 (11)	
N6	0.78483 (9)	0.84631 (6)	0.03858 (5)	0.01194 (10)	
C1	−0.17886 (12)	0.66190 (8)	0.55457 (6)	0.01753 (12)	
H1A	−0.0967	0.6423	0.5001	0.021*	
C2	−0.34245 (13)	0.58975 (8)	0.58220 (7)	0.02162 (14)	
H2A	−0.3682	0.5208	0.5470	0.026*	
C3	−0.46864 (12)	0.62002 (9)	0.66263 (7)	0.02278 (15)	
H3A	−0.5789	0.5721	0.6802	0.027*	
C4	−0.42941 (12)	0.72184 (8)	0.71643 (7)	0.02058 (14)	
H4A	−0.5135	0.7423	0.7698	0.025*	
C5	−0.26331 (11)	0.79319 (7)	0.68997 (6)	0.01674 (12)	
H5A	−0.2362	0.8604	0.7267	0.020*	
C6	−0.13718 (11)	0.76450 (7)	0.60870 (5)	0.01428 (11)	
C7	0.03747 (11)	0.83958 (7)	0.58273 (6)	0.01507 (12)	
H7A	0.0684	0.9026	0.6227	0.018*	
C8	0.40347 (10)	0.87300 (7)	0.40132 (5)	0.01289 (11)	
C9	0.52692 (13)	0.75797 (7)	0.23945 (6)	0.01832 (13)	
H9A	0.6125	0.6886	0.2826	0.022*	
H9B	0.4953	0.7268	0.1749	0.022*	
C10	0.63071 (10)	0.88567 (7)	0.20958 (5)	0.01271 (11)	
C11	0.70776 (10)	0.92958 (6)	0.10204 (5)	0.01202 (10)	
C12	0.70589 (10)	1.06188 (7)	0.03802 (5)	0.01354 (11)	
C13	0.82190 (10)	0.70199 (7)	0.06881 (5)	0.01254 (11)	
C14	0.73006 (11)	0.61939 (7)	0.01353 (6)	0.01455 (11)	
H14A	0.6507	0.6558	−0.0449	0.017*	
C15	0.76016 (11)	0.48012 (7)	0.04823 (7)	0.01743 (13)	
H15A	0.7000	0.4223	0.0128	0.021*	
C16	0.87966 (12)	0.42727 (7)	0.13554 (7)	0.01914 (13)	
H16A	0.8974	0.3343	0.1588	0.023*	
C17	0.97311 (13)	0.51258 (8)	0.18857 (7)	0.01997 (14)	
H17A	1.0547	0.4764	0.2461	0.024*	
C18	0.94424 (11)	0.65205 (7)	0.15537 (6)	0.01683 (12)	
H18A	1.0052	0.7101	0.1902	0.020*	
H1N3	0.607 (2)	0.9855 (15)	0.4163 (12)	0.029 (3)*	

### Atomic displacement parameters (Å^2^)

**Table:**

	*U*^11^	*U*^22^	*U*^33^	*U*^12^	*U*^13^	*U*^23^
S1	0.01861 (9)	0.02017 (9)	0.01455 (8)	−0.00900 (6)	0.00407 (6)	−0.00768 (6)
O1	0.0196 (2)	0.0137 (2)	0.0136 (2)	−0.00159 (18)	0.00192 (18)	−0.00089 (16)
O2	0.0219 (3)	0.0114 (2)	0.0231 (3)	0.00014 (18)	0.0021 (2)	−0.00294 (18)
N1	0.0161 (3)	0.0161 (2)	0.0132 (2)	−0.0050 (2)	0.00206 (19)	−0.00282 (18)
N2	0.0162 (3)	0.0164 (2)	0.0120 (2)	−0.0053 (2)	0.00244 (19)	−0.00370 (18)
N3	0.0157 (3)	0.0175 (2)	0.0113 (2)	−0.0056 (2)	0.00213 (19)	−0.00495 (18)
N4	0.0143 (2)	0.0144 (2)	0.0118 (2)	−0.00285 (18)	0.00189 (18)	−0.00367 (17)
N5	0.0180 (3)	0.0141 (2)	0.0126 (2)	−0.00152 (19)	0.00223 (19)	−0.00250 (18)
N6	0.0129 (2)	0.0116 (2)	0.0117 (2)	−0.00116 (17)	0.00069 (18)	−0.00305 (17)
C1	0.0191 (3)	0.0174 (3)	0.0166 (3)	−0.0043 (2)	−0.0004 (2)	−0.0035 (2)
C2	0.0211 (3)	0.0196 (3)	0.0245 (3)	−0.0070 (3)	−0.0030 (3)	−0.0028 (3)
C3	0.0163 (3)	0.0216 (3)	0.0282 (4)	−0.0053 (3)	−0.0003 (3)	0.0020 (3)
C4	0.0157 (3)	0.0206 (3)	0.0228 (3)	−0.0006 (2)	0.0038 (3)	0.0016 (3)
C5	0.0169 (3)	0.0153 (3)	0.0170 (3)	−0.0006 (2)	0.0025 (2)	−0.0010 (2)
C6	0.0153 (3)	0.0135 (2)	0.0135 (2)	−0.0024 (2)	0.0004 (2)	−0.0008 (2)
C7	0.0171 (3)	0.0151 (3)	0.0134 (3)	−0.0038 (2)	0.0023 (2)	−0.0029 (2)
C8	0.0150 (3)	0.0125 (2)	0.0114 (2)	−0.0027 (2)	0.0007 (2)	−0.00234 (19)
C9	0.0240 (3)	0.0139 (3)	0.0188 (3)	−0.0064 (2)	0.0084 (3)	−0.0063 (2)
C10	0.0145 (3)	0.0114 (2)	0.0126 (2)	−0.0022 (2)	0.0021 (2)	−0.00294 (19)
C11	0.0133 (3)	0.0109 (2)	0.0122 (2)	−0.00156 (19)	0.0016 (2)	−0.00314 (18)
C12	0.0138 (3)	0.0126 (2)	0.0142 (3)	−0.0017 (2)	0.0007 (2)	−0.0023 (2)
C13	0.0131 (3)	0.0111 (2)	0.0136 (2)	−0.00038 (19)	0.0008 (2)	−0.00321 (19)
C14	0.0138 (3)	0.0141 (3)	0.0168 (3)	−0.0015 (2)	0.0006 (2)	−0.0055 (2)
C15	0.0155 (3)	0.0136 (3)	0.0242 (3)	−0.0027 (2)	0.0037 (2)	−0.0061 (2)
C16	0.0182 (3)	0.0134 (3)	0.0244 (3)	0.0003 (2)	0.0049 (3)	−0.0012 (2)
C17	0.0205 (3)	0.0171 (3)	0.0203 (3)	0.0034 (2)	−0.0016 (3)	−0.0006 (2)
C18	0.0173 (3)	0.0162 (3)	0.0170 (3)	0.0009 (2)	−0.0030 (2)	−0.0037 (2)

### Geometric parameters (Å, °)

**Table:**

S1—C8	1.7400 (7)	C4—C5	1.3961 (11)
S1—C9	1.8126 (8)	C4—H4A	0.9300
O1—N5	1.3787 (8)	C5—C6	1.3996 (10)
O1—C12	1.4168 (9)	C5—H5A	0.9300
O2—C12	1.2120 (8)	C6—C7	1.4655 (10)
N1—C7	1.2835 (9)	C7—H7A	0.9300
N1—N2	1.3884 (9)	C9—C10	1.5022 (10)
N2—C8	1.3049 (9)	C9—H9A	0.9700
N3—C8	1.3689 (9)	C9—H9B	0.9700
N3—N4	1.3737 (8)	C10—C11	1.4567 (9)
N3—H1N3	0.852 (15)	C11—C12	1.4238 (9)
N4—C10	1.2940 (9)	C13—C14	1.3857 (10)
N5—N6	1.3110 (8)	C13—C18	1.3892 (10)
N6—C11	1.3553 (9)	C14—C15	1.3944 (10)
N6—C13	1.4418 (9)	C14—H14A	0.9300
C1—C2	1.3878 (11)	C15—C16	1.3898 (12)
C1—C6	1.4044 (10)	C15—H15A	0.9300
C1—H1A	0.9300	C16—C17	1.3940 (12)
C2—C3	1.3967 (13)	C16—H16A	0.9300
C2—H2A	0.9300	C17—C18	1.3935 (11)
C3—C4	1.3906 (13)	C17—H17A	0.9300
C3—H3A	0.9300	C18—H18A	0.9300
			
C8—S1—C9	96.96 (4)	N3—C8—S1	120.03 (5)
N5—O1—C12	111.40 (5)	C10—C9—S1	111.37 (5)
C7—N1—N2	115.29 (6)	C10—C9—H9A	109.4
C8—N2—N1	110.58 (6)	S1—C9—H9A	109.4
C8—N3—N4	125.97 (6)	C10—C9—H9B	109.4
C8—N3—H1N3	115.9 (11)	S1—C9—H9B	109.4
N4—N3—H1N3	111.4 (10)	H9A—C9—H9B	108.0
C10—N4—N3	118.01 (6)	N4—C10—C11	115.69 (6)
N6—N5—O1	103.91 (5)	N4—C10—C9	122.87 (6)
N5—N6—C11	115.66 (6)	C11—C10—C9	121.45 (6)
N5—N6—C13	118.02 (6)	N6—C11—C12	105.42 (6)
C11—N6—C13	126.28 (6)	N6—C11—C10	125.08 (6)
C2—C1—C6	120.13 (7)	C12—C11—C10	129.33 (6)
C2—C1—H1A	119.9	O2—C12—O1	120.16 (6)
C6—C1—H1A	119.9	O2—C12—C11	136.18 (7)
C1—C2—C3	120.37 (8)	O1—C12—C11	103.61 (5)
C1—C2—H2A	119.8	C14—C13—C18	122.93 (6)
C3—C2—H2A	119.8	C14—C13—N6	119.23 (6)
C4—C3—C2	120.01 (8)	C18—C13—N6	117.80 (6)
C4—C3—H3A	120.0	C13—C14—C15	118.00 (7)
C2—C3—H3A	120.0	C13—C14—H14A	121.0
C3—C4—C5	119.76 (8)	C15—C14—H14A	121.0
C3—C4—H4A	120.1	C16—C15—C14	120.34 (7)
C5—C4—H4A	120.1	C16—C15—H15A	119.8
C4—C5—C6	120.62 (7)	C14—C15—H15A	119.8
C4—C5—H5A	119.7	C15—C16—C17	120.51 (7)
C6—C5—H5A	119.7	C15—C16—H16A	119.7
C5—C6—C1	119.11 (7)	C17—C16—H16A	119.7
C5—C6—C7	119.85 (7)	C18—C17—C16	119.99 (7)
C1—C6—C7	121.04 (7)	C18—C17—H17A	120.0
N1—C7—C6	119.76 (6)	C16—C17—H17A	120.0
N1—C7—H7A	120.1	C13—C18—C17	118.21 (7)
C6—C7—H7A	120.1	C13—C18—H18A	120.9
N2—C8—N3	118.27 (6)	C17—C18—H18A	120.9
N2—C8—S1	121.63 (5)		
			
C7—N1—N2—C8	−173.31 (7)	N5—N6—C11—C12	0.31 (8)
C8—N3—N4—C10	−33.94 (10)	C13—N6—C11—C12	178.09 (6)
C12—O1—N5—N6	0.31 (8)	N5—N6—C11—C10	175.83 (7)
O1—N5—N6—C11	−0.39 (8)	C13—N6—C11—C10	−6.39 (11)
O1—N5—N6—C13	−178.36 (6)	N4—C10—C11—N6	145.60 (7)
C6—C1—C2—C3	1.12 (12)	C9—C10—C11—N6	−34.74 (11)
C1—C2—C3—C4	−0.80 (13)	N4—C10—C11—C12	−39.98 (11)
C2—C3—C4—C5	−0.21 (12)	C9—C10—C11—C12	139.68 (8)
C3—C4—C5—C6	0.90 (12)	N5—O1—C12—O2	−177.78 (7)
C4—C5—C6—C1	−0.58 (11)	N5—O1—C12—C11	−0.14 (8)
C4—C5—C6—C7	−179.42 (7)	N6—C11—C12—O2	176.97 (9)
C2—C1—C6—C5	−0.43 (11)	C10—C11—C12—O2	1.70 (14)
C2—C1—C6—C7	178.40 (7)	N6—C11—C12—O1	−0.09 (7)
N2—N1—C7—C6	−177.89 (6)	C10—C11—C12—O1	−175.35 (7)
C5—C6—C7—N1	−176.39 (7)	N5—N6—C13—C14	−62.37 (9)
C1—C6—C7—N1	4.79 (11)	C11—N6—C13—C14	119.90 (8)
N1—N2—C8—N3	−176.40 (6)	N5—N6—C13—C18	119.82 (7)
N1—N2—C8—S1	6.57 (9)	C11—N6—C13—C18	−57.91 (10)
N4—N3—C8—N2	−156.39 (7)	C18—C13—C14—C15	1.16 (11)
N4—N3—C8—S1	20.70 (10)	N6—C13—C14—C15	−176.54 (6)
C9—S1—C8—N2	−164.38 (6)	C13—C14—C15—C16	−0.22 (11)
C9—S1—C8—N3	18.64 (6)	C14—C15—C16—C17	−0.92 (12)
C8—S1—C9—C10	−45.98 (6)	C15—C16—C17—C18	1.17 (12)
N3—N4—C10—C11	175.86 (6)	C14—C13—C18—C17	−0.91 (11)
N3—N4—C10—C9	−3.80 (11)	N6—C13—C18—C17	176.81 (7)
S1—C9—C10—N4	45.22 (9)	C16—C17—C18—C13	−0.27 (12)
S1—C9—C10—C11	−134.42 (6)		

### Hydrogen-bond geometry (Å, °)

**Table:**

*D*—H···*A*	*D*—H	H···*A*	*D*···*A*	*D*—H···*A*
N3—H1N3···N2^i^	0.852 (15)	2.015 (15)	2.8664 (9)	178.3 (11)
C14—H14A···O2^ii^	0.93	2.58	3.2303 (10)	127
C15—H15A···O2^iii^	0.93	2.51	3.2391 (9)	136
C18—H18A···S1^iv^	0.93	2.84	3.7061 (8)	155

Symmetry codes: (i) −*x*+1, −*y*+2, −*z*+1; (ii) −*x*+1, −*y*+2, −*z*; (iii) *x*, *y*−1, *z*; (iv) *x*+1, *y*, *z*.
